# Distal Clavicle Osteolysis in a 30-Year-Old Male: A Case Report

**DOI:** 10.7759/cureus.84697

**Published:** 2025-05-23

**Authors:** Joshua L Dale, Kip Owen

**Affiliations:** 1 Osteopathic Medicine, William Carey University College of Osteopathic Medicine, Hattiesburg, USA; 2 Orthopaedic Surgery, Doctors Hospital at Renaissance, Edinburg, USA

**Keywords:** amateur athlete, distal clavicular osteolysis, general radiology, medical education, orthopedic sports medicine

## Abstract

Distal clavicle osteolysis (DCO) is an overuse injury characterized by pain and bone resorption at the acromioclavicular (AC) joint due to repetitive microtrauma. Early diagnosis is critical to prevent prolonged symptoms and functional impairment, as conservative treatment is most effective when initiated promptly. We present the case of a 30-year-old male who initially injured his right shoulder during a fall in judo and reinjured it while lifting a patient. He reported persistent AC joint pain exacerbated by overhead activities and wearing a lead apron during surgery. Physical examination revealed localized AC joint tenderness with positive Jobe, Speed, and Cross Arm tests. Imaging studies included X-rays showing AC joint widening and MRI findings consistent with DCO. Conservative management - rest, activity modification, and range-of-motion exercises - was initiated, and pharmacological or surgical interventions were deferred. The patient experienced ongoing functional limitations due to delayed diagnosis, initially being misdiagnosed with rotator cuff tendonitis. With a formal diagnosis of DCO, conservative treatment was implemented. The patient opted to defer corticosteroid injections or the Mumford procedure but was counseled on these options should symptoms persist. This case highlights the importance of early and accurate diagnosis of DCO to prevent unnecessary suffering and optimize outcomes. Timely imaging and a thorough clinical assessment are essential for distinguishing DCO from other shoulder pathologies. Conservative management remains effective if initiated early, reducing the likelihood of surgical intervention and improved patient outcomes.

## Introduction

The acromioclavicular (AC) joint is a diarthrodial joint formed at the intersection of the acromion process and lateral end of the clavicle and medial portion of the acromion [1,2]. Distal clavicle osteolysis (DCO), often known as "weight lifters' shoulder," is typically an overuse injury observed in athletes and weightlifters, characterized by pain localized to the AC joint [1]. Activities that contribute to DCO involve repetitive and excessive loading of the AC joint, often through motions such as horizontal abduction, adduction, internal rotation, and forward or lateral flexion [1].

AC joint pathology generally arises from one of three processes: trauma (e.g., fracture, AC joint separation, dislocation), AC joint arthrosis (post-traumatic or idiopathic), or DCO [2]. Static stability of the AC joint is provided by the coracoclavicular ligaments, while dynamic stabilization is maintained by the deltoid and trapezius muscles. The primary role of the acromioclavicular ligament is to connect the clavicle to the scapula, thereby controlling the movement of both structures.

The most common mechanism of AC joint injury involves a direct force applied to the superior aspect of the shoulder while the arm is in an adducted position [2]. Injuries and sprains to the AC joint are classified using the Rockwood classification system, which grades them on a scale from 1 to 3. This system evaluates four components: the acromioclavicular ligament, coracoclavicular ligament, and the deltoid and trapezius musculature. Grade I represents a sprain of the AC ligament with no involvement of other ligaments or musculature; Grade II involves a complete tear of the AC ligament, disruption of the AC joint, a sprain of the coracoclavicular ligament, and possible detachment of the deltoid and trapezius muscles; Grade III features a complete tear of the AC ligament, dislocation of the AC joint with superior displacement of the clavicle relative to the acromion, disruption of the coracoclavicular ligament with a widened interval of up to 100%, and a high probability of deltoid and trapezius detachment from the distal clavicle [3].

This case involves an adult male who fell onto his right shoulder while engaging in sports activities. The patient is actively following up with the clinic.

## Case presentation

The patient is a 30-year-old male who presented to the clinic with complaints of right shoulder pain. He reported initially injuring his shoulder six months ago while teaching a judo class. The patient mentioned that he teaches judo recreationally, while his primary occupation is in the hospital. He explained that during a maneuver, he fell directly onto his shoulder with his arm tucked into his side. Following the injury, the patient experienced some relief after one to two months but subsequently reinjured the same shoulder while lifting a heavy patient at the hospital. Since the second injury, he has experienced persistent pain, particularly with excessive overhead motion. He also reports radiation of pain into the belly of the supraspinatus muscle, especially when wearing a lead apron in the operating room (OR). Approximately one month ago, the patient sought medical attention from another physician and was diagnosed with rotator cuff tendonitis. He was prescribed Celebrex (a non-steroidal anti-inflammatory drug (NSAID)) 200 mg daily for a month, which he took as directed, but he reported no significant relief. He has now presented for a second opinion.

Medical history

The patient's medical history is significant for hypertension, which is controlled with losartan and amlodipine. He reports only two traumatic incidents: the initial shoulder injury during judo and the subsequent reinjury while lifting a heavy patient. He denies any history of smoking, recent alcohol use, or anticoagulant therapy that could affect musculoskeletal treatment. Surgical history includes an adenoidectomy performed during childhood. He reports weakness and pain with motion and resistance of the right upper extremity but denies any other musculoskeletal complaints, fever, or systemic symptoms.

Physical exam findings

The patient presented with the following vital signs: blood pressure (BP) of 144/60 mmHg, heart rate of 55 bpm, respiratory rate of 10 breaths per minute, and oxygen saturation of 98% on room air. The patient’s body mass index (BMI) was calculated at 32. A neurological examination revealed symmetric reflexes graded at 2+ in the biceps, triceps, and brachioradialis bilaterally. Strength testing demonstrated 5/5 strength bilaterally in the upper extremities, including the wrist flexors, extensors, and shoulder abductors. Sensation was intact across all dermatomes with no evidence of diminished perception. Passive and active range of motion (ROM) were symmetrical bilaterally. Shoulder flexion was 180°, extension 60°, abduction 180°, adduction 30°, external rotation 90°, internal rotation 70°, horizontal abduction 45°, and horizontal adduction 135°. Range of motion testing is summarized in Table 1. The following orthopedic tests were performed during this encounter: Jobe test, Speed test, Hawkins test, Cross Arm test, Hornblower’s test, Belly Press test, and Lift-Off test. Positive findings were noted for the Jobe test, Speed test, and Cross Arm test on the right shoulder. All other special tests did not elicit pain or symptomatology. Special test results as well as descriptions of tests are summarized in Table 2.

**Table 1:** Summary of Range of Motion Testing

Movement	Normal Range (Degrees)	Patient Range (Degrees)
Flexion	0 – 180°	180°
Extension	0 – 60°	60°
Abduction	0 – 180°	180°
Adduction	0 – 30°	30°
External Rotation	0 – 90°	90°
Internal Rotation	0 – 70°	70°
Horizontal Abduction	0 – 45°	45°
Horizontal Adduction	0 – 135°	135°

**Table 2:** Summary of Special Tests Conducted with Method and Patient Result AC: acromioclavicular

Test Name	Method	Patient Result
Jobe Test (Empty Can)	Patient’s arm abducted to 90°, internally rotated with slight forward flexion; resists downward pressure	Positive (Right Shoulder)
Speed Test	Patient’s arm extended and supinated, shoulder flexed to 90°; resists downward force	Positive (Right Shoulder)
Cross Arm Test	Patient’s arm flexed to 90° and adducted across chest; pain at AC joint indicates positive test	Positive (Right Shoulder)
Hornblower's Test	Patient’s arm elevated to 90° in scapular plane, elbow flexed to 90°; externally rotates against resistance	Negative
Belly Press Test	Patient presses hand into abdomen with elbow forward; inability to maintain indicates weakness	Negative
Lift-Off Test	Patient places hand behind back and attempts to lift it off; inability indicates weakness	Negative
Hawkins-Kennedy Test	Patient’s arm and elbow flexed to 90°; examiner internally rotates shoulder; pain during rotation indicates positive test	Negative

Imaging and labs

**Figure 1:**
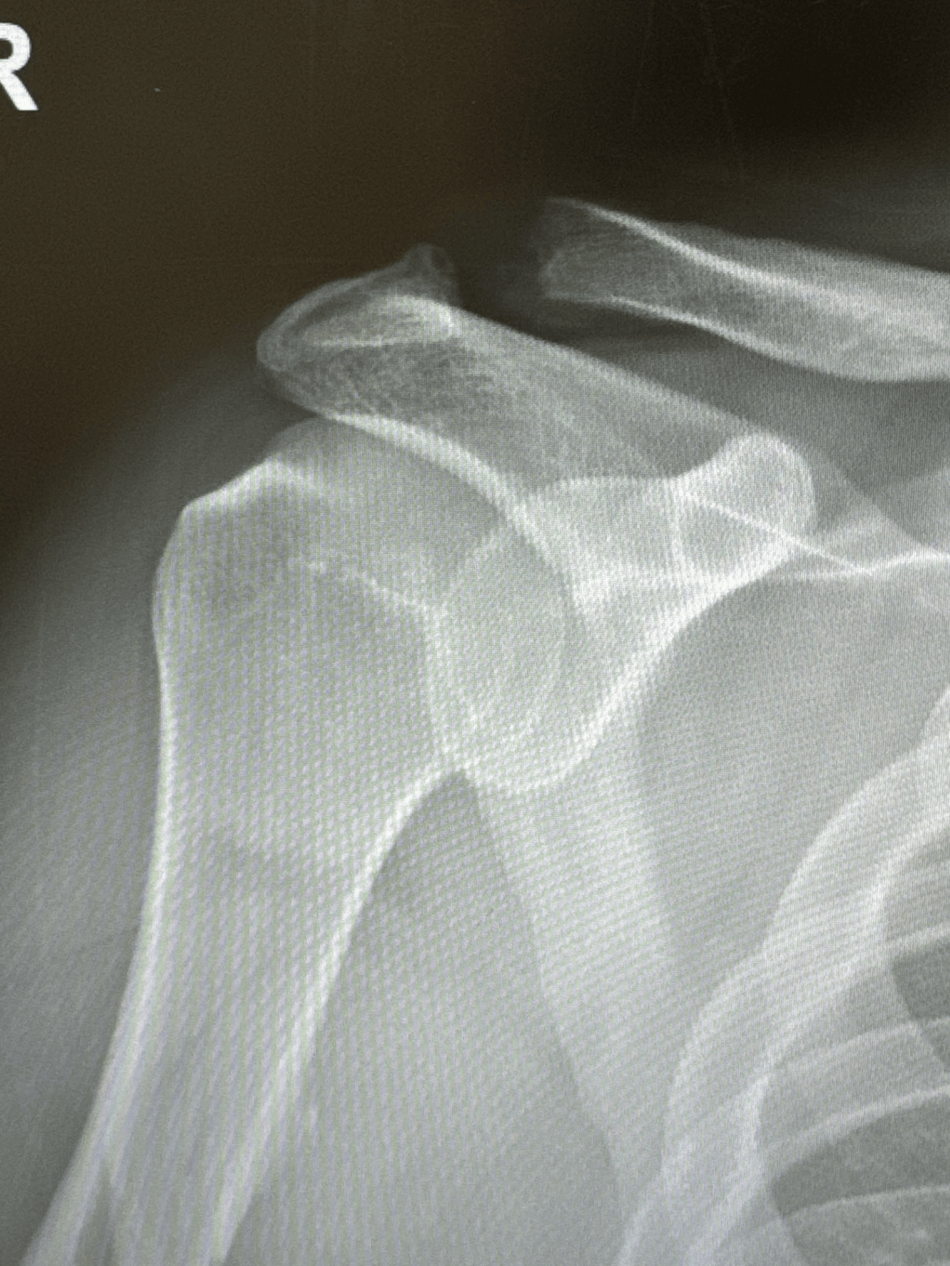
Anteroposterior (AP) right shoulder

Patient received an X-ray at our initial orthopedic consult, which demonstrated widening at the AC joint. Initial X-ray presented an anteroposterior (AP) view shown in Figure 1. 

As there was a concern for soft tissue damage, an MRI was ordered and completed. The results of the MRI demonstrated widening of the AC joint suggestive of distal clavicle surgical excision.

**Figure 2:**
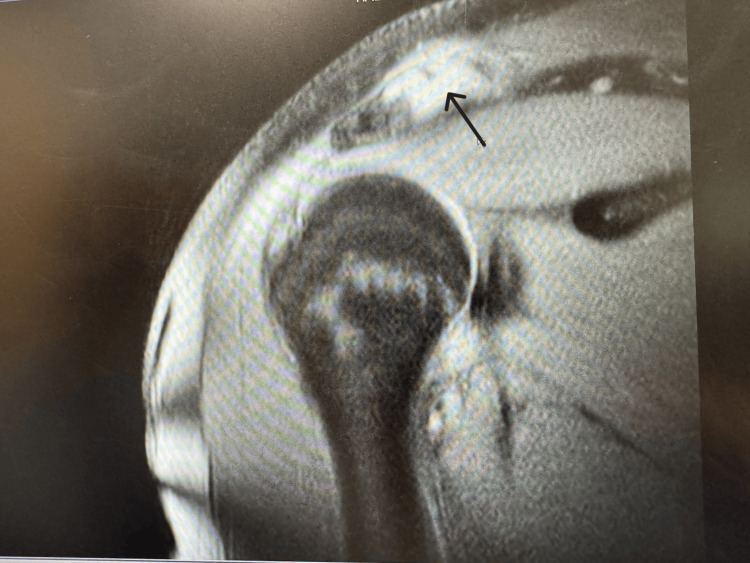
Coronal view right shoulder initial consult Arrow pointing towards area of osteolysis of the distal clavicle

The MRI of the right shoulder demonstrates no evidence of fractures or dislocation. The glenohumeral joint space is preserved, and the acromioclavicular joint shows signs of hyperlucency and some degree of separation. The humeral head is normally aligned within the glenoid fossa. The rotator cuff, including the supraspinatus, infraspinatus, subscapularis, and teres minor tendons, is intact with normal signal intensity and thickness. There is no evidence of tendinopathy, tears, or atrophy. The glenoid labrum appears intact without signs of tears or detachment. The glenohumeral ligaments and coracohumeral ligaments are normal in appearance, with no evidence of tears or laxity. The subacromial-subdeltoid bursa is normal in size with no fluid accumulation or inflammation. Articular cartilage of the glenohumeral and acromioclavicular joints is well-preserved, showing no degeneration or defects. The peri-scapular and rotator cuff muscles demonstrate normal bulk and signal intensity, with no signs of muscle atrophy or edema. The brachial plexus and adjacent neurovascular structures are normal, with no masses or compressive lesions identified. Surrounding soft tissues are unremarkable, with no fluid collections or abnormalities. The MRI image is shown in Figure 2. Given the MRI report, the patient would be a Grade I on the Rockwood classification, with clavicular involvement. 

Therapeutic intervention

For pain relief, the patient was counseled on the following options: no intervention, oral NSAIDs, corticosteroid injection, or Mumford surgery. The patient expressed that, since Celebrex had not provided relief in the past, he preferred to continue range of motion exercises at home without the need for oral medication or injections. He was advised to restrict lifting to weights under 20 lbs. for the next three months and was provided with a work note for protective purposes. The patient was informed that if pain persists despite the work restriction, he may require an injection or Mumford surgery for symptom relief. The patient agreed to schedule a follow-up appointment in three months for repeat X-rays.

**Figure 3:**
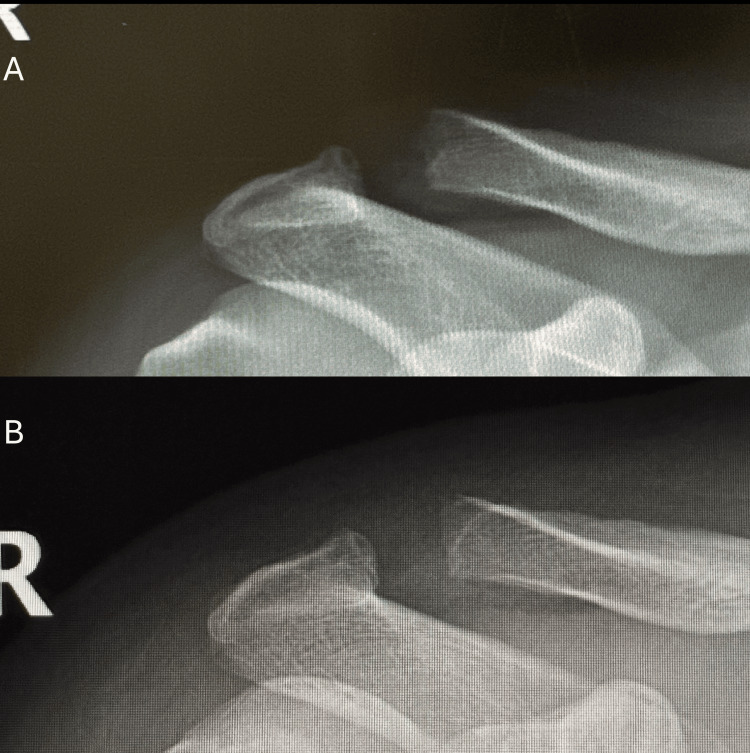
Anteroposterior (AP) right shoulder three-month follow-up A is patient's clavicle at initial consult B is patient's clavicle at three-month follow-up

The patient successfully underwent repeat imaging at the three-month follow-up. The imaging demonstrated cessation of osteolysis of the distal clavicle, with evidence of cortical smoothing and remodeling of the bony margins. Comparative radiographs, with the initial image presented above and the three-month follow-up below, are shown in Figure 3.

## Discussion

DCO is a condition typically seen in athletes or individuals who engage in repetitive overhead motions [1]. It is characterized by pain in the distal clavicle and gradual bone resorption due to repetitive microtrauma. The AC joint, a highly mobile structure, plays a critical role in providing both stability and mobility to the shoulder girdle, allowing for overhead movements [4]. The joint's stability is supported by multiple ligaments: the AC ligament, which consists of superior, inferior, and posterior bundles, and the coracoclavicular (CC) ligaments (conoid and trapezoid) [4]. The AC ligaments contribute to joint stability by controlling superior displacement, as well as horizontal and vertical stability, while the CC ligaments are considered the primary stabilizers of the joint, providing superior and inferior stability compared to the AC ligaments [5]. As osteolysis progresses, the patient experiences increased pain and difficulty with overhead motions, as was the case with the patient in this report.

AC joint pathology typically arises from one of three etiologies: trauma, osteoarthritis, or DCO [2]. AC joint separations are a common cause of shoulder injury, usually resulting from a fall or direct force applied to the superior aspect of the shoulder with the arm in an adducted position, which is similar to the injury reported by the patient [2]. These injuries are graded using the Rockwood classification system, as summarized in Table 3. The patient's initial X-rays indicated a Rockwood Grade 1 diagnosis, with pain around the AC joint without weakness or involvement of other structures. The patient reported relief about a month after the initial injury, during which he rested the shoulder but did not seek professional medical care. He resumed working with patients once he felt better, which he attributes to reinjuring the shoulder about six weeks later. After this second injury, the patient continued his activities despite significant pain, which aligns with the development of DCO. The most accepted etiology of DCO involves repetitive microfractures of the subchondral bone, leading to attempts at repair [6]. Patients with DCO typically present with an insidious onset of AC joint pain, aggravated by cross-body adduction movements and weight training [6].

**Table 3:** Summary of Rockwood Classification AC: acromioclavicular

Grade	AC Ligament	AC Joint	Coracoclavicular Ligament	Deltoid & Trapezius	Clavicle Position
Grade 1	Sprain (partial injury)	Intact	Intact	Unaffected	Normal
Grade 2	Complete tear	Disrupted	Sprain	Possible detachment	Normal
Grade 3	Complete tear	Dislocated, clavicle displaced	Disrupted (widened interval)	High probability of detachment	Superiorly displaced compared to acromion

The association between weightlifting and DCO is well established [6,7]. Torrence et al. examined a group of individuals diagnosed with DCO and found that 81% of cases were attributed to sports or weightlifting, while approximately 5% resulted from a fall, as seen in our patient [7]. Work-related factors may also contribute to the development of DCO [6]. For instance, DCO is frequently observed in manual laborers whose jobs involve heavy overhead lifting, such as builders or plasterers [6]. Repetitive microtrauma in the area can generate debris, which stimulates macrophages to release inflammatory cytokines, including tumor necrosis factor-alpha (TNF-α), interleukin (IL)-1β, and IL-6 [8]. TNF-α and IL-6 are known to be catabolic to bone, while IL-1β promotes osteoclast differentiation, leading to bone resorption [8]. In this case, the patient initially sustained an injury from a fall but continued to engage in work-related activities and weightlifting throughout the recovery period. These combined factors likely contributed to the development of their DCO.

The patient's positive Jobe and Speed tests suggested potential involvement of the supraspinatus and the biceps/labral complex [9,10]. Given the positive orthopedic test findings and overlapping symptoms, an MRI was ordered to rule out rotator cuff involvement. The MRI revealed no abnormalities apart from signs of involvement in the AC joint. The patient presented with a unique symptom pattern, including pain exacerbated by wearing a lead apron, which prompted his visit for a second opinion. The pain radiated primarily into the belly of the supraspinatus muscle, likely due to inflammation surrounding the AC joint that mimicked supraspinatus tendon impingement symptoms [11]. Previously, the patient had been diagnosed with rotator cuff tendonitis and was not prescribed further imaging. This highlights the critical importance of appropriate imaging in establishing an accurate diagnosis.

In most patients, plain film radiographs are sufficient to diagnose DCO, typically revealing microcysts, subchondral bone loss, and osteolysis of the distal clavicle [1]. However, if plain radiographs are inconclusive, as in our patient, advanced imaging modalities such as MRI, bone scan, or ultrasound can be utilized to establish a more definitive diagnosis [1]. For patients presenting with symptoms resembling a superior labrum anterior to posterior (SLAP) tear, an MRI may be particularly beneficial in providing a thorough evaluation [1]. Based on the patient's imaging, history, and physical exam findings, a formal diagnosis of DCO was made.

The preferred approach to treatment is typically conservative, involving rest, NSAIDs, local anesthetics, kinesiology tape, and physical therapy [12]. In a study by Mestan et al., four out of seven patients reported improvement with conservative management of DCO [13]. Proper management that showed success in other reports demonstrated a protocol of strict rest from overhead activities followed by rehab and manual therapy, with the patient resolving symptoms in six months with improved ranges of motion and activities of daily living [14]. The patient expressed a preference for continuing therapy at home and declined a formal physical therapy prescription. However, if conservative measures fail to show improvement, surgical intervention may be required [14]. The most performed surgery for DCO is the Mumford procedure, which involves resecting the distal clavicle. This procedure has been shown to be effective in treating post-traumatic degenerative disease of the AC joint, distal clavicle, and impingement syndrome [15]. The patient is scheduled for a follow-up appointment in three months for repeat X-rays. If there is no improvement in pain or X-ray findings, the Mumford procedure will be discussed as a potential next step.

The Mumford procedure can be performed using either an open or arthroscopic approach [15,16]. Novak et al. reported significant success with the open technique [16]. In Novak's study, patients were interviewed for 30 months postoperatively to evaluate outcomes [16]. The patients were assessed using the Hospital for Special Surgery (HSS) criteria, where scores were categorized as excellent (90-100), good (70-89), fair (50-69), or poor (<50) [16]. Of the 23 patients interviewed, 18 achieved good or excellent postoperative ratings, regardless of age [16]. These patients also demonstrated improved range of motion and a reduction in pain along the distal clavicle [16]. The arthroscopic approach also shows high levels of success and offers additional benefits, such as the ability to address concomitant rotator cuff tears [15]. Lesko studied 57 patients, 39 of whom had concurrent rotator cuff tears [15]. Among this group, all 57 patients reported significant improvement in distal clavicular pain following the procedure [15]. Patients who undergo arthroscopic distal clavicle osteolysis are likely to experience statistically and clinically significant improvements in passive range of motion and active range of motion [17].

## Conclusions

DCO, often referred to as "weightlifter’s shoulder," is an overuse injury that is particularly common in athletes. The condition is characterized by pain around the AC joint, primarily caused by repeated microtrauma. Early identification is essential for effective treatment, allowing the patient to initiate conservative measures such as rest, physical therapy, NSAIDs, and corticosteroid injections. If conservative treatments fail, the Mumford procedure, which involves partial resection of the distal clavicle, is the recommended option for long-term symptom relief.

This case highlights the importance of obtaining a proper diagnosis early. The patient neglected treatment for an extended period and, when seeking care, was initially given an incorrect diagnosis. With earlier detection, the patient may have experienced resolution of symptoms much sooner, rather than suffering for six months. The patient is scheduled for a follow-up appointment in three months, at which time further evaluation will be conducted and a future treatment plan will be determined.
